# Physical Activity and Mortality in Patients With Chronic Kidney Disease: A Protocol for Systematic Review and Dose-Response Meta-Analysis

**DOI:** 10.3389/fmed.2022.861013

**Published:** 2022-04-11

**Authors:** Fan Zhang, Hui Wang, Liuyan Huang, Yan Bai

**Affiliations:** ^1^Department of Nephrology, Longhua Hospital Shanghai University of Traditional Chinese Medicine, Shanghai, China; ^2^Department of Anorectology, Longhua Hospital Shanghai University of Traditional Chinese Medicine, Shanghai, China; ^3^Department of Cardiology, Longhua Hospital Shanghai University of Traditional Chinese Medicine, Shanghai, China

**Keywords:** physical activity, chronic kidney disease, mortality, dose-response, meta-analysis

## Abstract

**Objective:**

To examine the dose-response associations between total physical activity, different intensity of physical activity, and all-cause mortality in patients with chronic kidney disease (CKD).

**Methods and Analysis:**

PubMed, Embase, Web of Science, and the Cochrane library will be searched from inception to June 2022. Only cohort studies assessing physical activity associations with all-cause mortality among CKD patients will be considered for inclusion. The quality of included cohort studies will be evaluated according to the Newcastle-Ottawa Scale (NOS). The robust error meta-regression (REMR) model will be used to establish dose-response relationships between physical activity and mortality. Additional statistical analysis including Egger's test, subgroup analysis, sensitivity analysis. The strengths of evidence will be evaluated with the Grading of Recommendation, Assessment, Development, and Evaluation approach.

**Ethics and Dissemination:**

Ethics approval is not required as no private information from individuals is collected.

**PROSPERO Registration Number:**

CRD 42021283630.

## Introduction

Chronic kidney disease (CKD) is a progressive lifelong disease that affects ~750 million people worldwide, and more than two million people have end-stage renal disease and require renal replacement therapy to stay alive (1, 2). In recent years, the life expectancy of CKD patients has increased with advances in dialysis or kidney transplantation techniques. Nevertheless, due to cardiovascular disease in more than 50% of CKD patients, the risk of premature death in CKD patients is 20 times higher than in the general population (3), placing a burden on individuals, families, and healthcare systems (4).

Physical activity is any physical movement produced by skeletal muscle that requires energy expenditure (5). Physical activity is the cornerstone of renal rehabilitation management in patients with CKD (6). There is evidence that even small increases in physical activity levels can improve exercise tolerance and cardiovascular responsiveness and improve quality of life in patients with CKD (7).

Intriguingly, a negative association between physical activity and all-cause mortality has been validated in the elderly (8) and other chronic disease populations such as cancer (9). To date, two meta-analyses have aimed to determine the association between physical activity and mortality risk in patients with CKD. These included a low number of studies (up to 11) or focused only on a certain stage of the CKD population (e.g., predialysis CKD or end-stage renal disease) (10, 11). Nevertheless, a dose-response relationship between physical activity levels and patients with full-spectrum CKD has not been established.

For this reason, we proposed to perform a dose-response meta-analysis of a cohort study of physical activity, all-cause mortality risk in CKD patients (including predialysis, peritoneal dialysis, hemodialysis, and kidney transplant recipients). We aim to elucidate the strength of this association, the shape of the dose-response relationship, potential sources of heterogeneity between studies, and the difference across activity domains.

## Methods

This systematic review and meta-analysis will be performed according to the Meta-analysis of Observational Studies in Epidemiology (MOOSE) guideline (12). The protocol for the systematic review has been registered with the International Prospective Register of Systematic Reviews (CRD 42021283630).

### Eligibility Criteria

Studies meeting the following inclusion criteria will be included: (1) patients aged over 18 years with chronic kidney disease and predialysis, peritoneal dialysis, hemodialysis, kidney transplant recipients will be eligible. (2) The relationship between physical activity and all-cause mortality outcomes in patients with CKD was reported; (3) adjusted effect sizes [hazard ratios (HRs), relative risks (RRs), odd ratios (ORs)] and corresponding 95% confidence intervals (CI) are provided; (4) reported physical activity doses in a metabolic equivalent task (MET)-h/week or other measures that can be used to calculate analytical values. (5) The study design was a cohort study. Exclusion criteria: (1) protocol, review, or without adequate information for analysis; (2) written in non-English.

### Search Strategy

This systematic review and meta-analysis will retrieve potential studies in the PubMed, Embase, Web of Science, and the Cochrane Library database based on keywords and medical subject heading (MeSH) terms such as “physical activity,” “chronic kidney disease,” and “mortality.” The detailed search strategy is listed in Supplementary File 1. The retrieval time is from inception to June 2022. To reduce the possibility of missing searches, we considered including “exercise” as a similar term to “physical activity.” In addition, we will also check references to similar systematic reviews to further identify potential studies.

### Literature Selection

**Figure 1:**
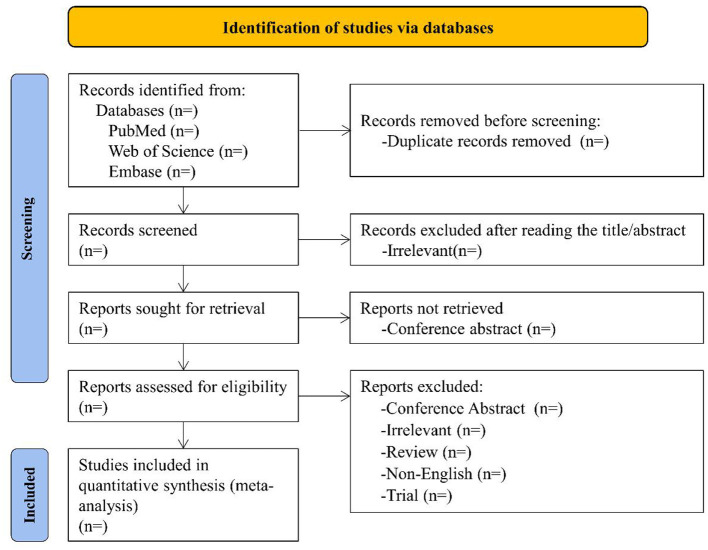
Flowchart showing the results of the selection process.

Two authors (YB and HW) will perform the literature selection, and a third author (LH) will help resolve any discrepancies if encountered. First, the titles and abstracts of all retrieved articles will be independently reviewed, and studies that do not meet the inclusion criteria will be excluded. Then, to determine if the articles meet the inclusion criteria, each author will further review the full text of the remaining studies. The flowchart of the literature search and selection strategy is shown in Figure 1.

The Cohen kappa statistic (κ) was calculated during the screening process to assess the level of agreement of reviewers. Consistency was classified a priori as follows: substantial consistency: κ > 0.60; moderate consistency: 0.21 ≤ κ ≤ 0.60; slight consistency: κ < 0.21 (13).

### Data Extraction and Risk of Bias

Two authors (YB and HW) will extract data according to Table 1. Disagreements will be resolved by consensus or discussion with a third author (LH). The relevant data to be collected include baseline characteristics of the population (e.g., study design, data source, age, gender, and sample size), stage of CKD, number of deaths, follow-up time, physical activity measures, physical activity units (e.g., kilocalories/week, MET^*^h/week, or steps/day), covariates from multivariate models, and adjusted effect sizes (ORs/RRs/HRs) and 95% CI in each physical activity category. A total effect size will be collected if two or more adjusted effect sizes were reported in a study. This study proposes to use the Newcastle-Ottawa Scale (NOS) to assess the quality of cohort studies. The scale has three domains with a maximum possible score of nine: selection of the cohort (0–4), comparability of the cohort (0–2), and assessment of the outcome (0–3). A NOS score of ≥6 is considered medium to high quality. Otherwise, it is considered low quality.

**Table 1:** The characteristic of included studies.

**References**	**Location; No of Participants; No of cases**	**Mean follow-up**	**Case ascertainment**	**Physical activity measurement**	**Covariates**	**NOS**
Author 1						
Author 2						
Author 3						
…						

### Exposure Quantification

First, we harmonized the group-level exposure estimates in MET^*^h/week to pool physical activity of different intensities and durations accumulated over the week. Physical activity was classified as low intensity (<3 METs), moderate-intensity (3–5.9 METs), and vigorous-intensity (≥6 METs) according to WHO classification criteria (14). When a study reported separate but uncombined results for different subgroups, we will use a fixed-effects model to combine the results for specific subgroups to obtain an overall estimate for the primary analysis. For studies that did not use the lowest category of physical activity as the reference category, we will recalculate the RRs using the method of Hamling et al. so that the lowest category becomes the reference (15).

When a study did not directly report physical activity, it was calculated by multiplying the median or midpoint duration of the reporting category with its assigned total MET value. The median or mean of the physical activity in each category was assigned to the corresponding RR for each study. For studies that reported physical activity, we estimated the midpoint of each category by calculating the mean of the lower and upper bounds. When the highest or lowest category was open-ended, we assumed that the length of the open interval was the same as the adjacent interval. For studies that reported physical activity at a weekly or monthly frequency, we converted frequency to hours per week or month by setting the dose of each activity at 45 min. If only the average duration of physical activity (e.g., walking) was reported, we assumed intensity of 4.5 METs. In addition, when studies reported kcal/week, we would convert between kcal/week (Y) and MET-h/week (X) using the following equation: X (MET^*^h)/Y (kcal) = 4.5 MET^*^2.5 h/550 kcal (16). The calculation of the dose distribution is shown in Table 2.

**Table 2:** Summary of MET*h/week assignment calculations for included studies.

**References**	**Physical activity type**	**Frequency**	**Duration**	**Intensity**	**RRs (95% CI)**	Assigned MET*h/week
Author 1						
Author 2						
Author 3						
…						

In sensitivity analyses, the intensity of marginalized PA doses (MET-h/week) was considered by subtracting the resting metabolic rate of 1 MET from the original mean PA intensity (2, 3, 3.5, and 7 MET for mild physical activity, moderate physical activity, moderate to vigorous physical activity, and vigorous physical activity, respectively).

## Data Synthesis

### Meta-Analysis

The primary outcome of this systematic review and dose-response meta-analysis will be all-cause mortality, and the secondary outcome will be cardiovascular mortality.

We will use a random-effects model to calculate the pooled RR and 95% CI for the highest vs. lowest physical activity level. We then used a robust error meta-regression (REMR) model to establish dose-response relationships between physical activity and mortality (17). The REMR model is a one-stage procedure that treats each study as a cohort and weights the effects of each study by its inverse variance while using robust variance to address the potential correlation of within-study effects. Considering that the dose-response relationship may be a non-linear pattern, we will use a restricted cubic spline function to fit the dose-response curve (18). This was done by setting three fixed nodes on the 5, 50, and 95 quartiles (19). The Wald test was used to test for non-linearity, with the null hypothesis that the coefficient of the non-linear term is equal to zero. All analyses will be carried out in Stata 12.0, and *P* < 0.05 was considered statistically significant.

### Heterogeneity

Given the potential heterogeneity, a random effect model will assess the summary effect sizes. We will quantify statistical heterogeneity using the *I*^2^ statistic. The evaluation criteria are as follow: <25% means small; 25–50% represents moderate heterogeneity; ≥50% considers heterogeneity. Subgroup analysis and meta-regression were used to explore potential heterogeneity based on specific characteristics, such as region, follow-up time, gender, sample size, and disease stage. Funnel plots and Egger's tests will be conducted to investigate publication bias across studies.

### Subgroup Analyses

If adequate data are available, we will perform subgroup analysis for the following: (1) region; (2) physical activity of different intensity (low-moderate vs. vigorous); (3) CKD stage (pre-dialysis vs. peritoneal dialysis vs. hemodialysis vs. kidney transplant); (4) follow-up duration [short term (1–3 months) vs. medium term (3–12 months) vs. long term (≥1 year)].

### Sensitivity Analysis

Sensitivity analysis will be performed to test the stability of the meta-analysis result, such as examining the effect of individual studies on the overall effect size by omitting one study.

### Quality of Evidence

We will use the Grading of Recommendations, Assessment, Development and Evaluation (GRADE) approach (study limitations, consistency of effect, imprecision, indirectness, and publication bias) to determine the outcome's overall quality of the evidence (20). Furthermore, a summary of the findings table will be developed using the GRADEpro Tool (https://gradepro.org/).

## Discussion

### Clinical Applications

Increasing physical activity levels may lead to improved physical function and, in turn, maintenance of independence and improved survival rates in patients with CKD (7). Therefore, most guidelines recommend 150 min of moderate intensity physical activity per week for CKD (21, 22). However, this recommendation is based on results from the general population, and the level of physical activity that would benefit people with chronic disease who have CKD is still unknown. The results of this study will address such clinical evidence.

## Limitation

This meta-analysis will present evidence related to all-cause mortality and physical activity in CKD patients. Nevertheless, some potential limitations need to be noted. First, different disease stages, gender, race, and other confounding factors may cause more significant heterogeneity in the results; therefore, if sufficient data are available, we will identify sources of heterogeneity through subgroup analysis and meta-regression and determine the stability of the results through sensitivity analysis. Second, the method of calculating physical activity dose according to the intensity or duration of physical activity exposure may distort the proper relationship between physical activity level and all-cause mortality. Third, the included studies contained self-reported physical activity, which may be prone to recall bias and overestimate effect values.

## Author Contributions

LH: conceptualization and writing—review and editing. FZ and YB: methodology. FZ and HW: writing—original draft. All authors contributed to the article and approved the submitted version.

## Funding

This study was supported by Shanghai Key Clinical Specialty Construction Project-Chinese Medicine Nephropathy (Grant No: shslczdzk04201) and Longhua Hospital Shanghai University of Traditional Chinese Medicine (Grant No: YW.006.035).

## Conflict of Interest

The authors declare that the research was conducted in the absence of any commercial or financial relationships that could be construed as a potential conflict of interest.

## Publisher's Note

All claims expressed in this article are solely those of the authors and do not necessarily represent those of their affiliated organizations, or those of the publisher, the editors and the reviewers. Any product that may be evaluated in this article, or claim that may be made by its manufacturer, is not guaranteed or endorsed by the publisher.

## Supplementary Material

Supplementary File1 Search detail for the database.
