# Supplementary material for: Physical Activity and Mortality in Patients With Chronic Kidney Disease: A Protocol for Systematic Review and Dose-Response Meta-Analysis
Source: Front Med (Lausanne). 2022 Apr 11;9:861013. doi: 10.3389/fmed.2022.861013 (PMC9063632; doi:10.3389/fmed.2022.861013)
Supplement: Supplementary File1 — Search detail for the database. [file Data_Sheet_1.pdf]

PubMed

(“mortality”[Mesh Terms] OR “mortalities”[Title/Abstract] OR “mortality”[Title/Abstract] OR “fatality”[Title/Abstract] OR “fatal”[Title/Abstract] OR “death”[Title/Abstract]) AND ("predialysis"[Title/Abstract] OR "pre-dialysis"[Title/Abstract] OR "kidney disease"[Title/Abstract] OR "renal disease"[Title/Abstract] OR "kidney failure"[Title/Abstract] OR "renal failure"[Title/Abstract] OR "hemodialysis"[Title/Abstract] OR "haemodialysis"[Title/Abstract] OR "hemofiltration"[Title/Abstract] OR "haemofiltration"[Title/Abstract] OR "hemodiafiltration"[Title/Abstract] OR "haemodiafiltration"[Title/Abstract] OR "dialysis"[Title/Abstract] OR "Renal Insufficiency"[MeSH Terms] OR "Renal Replacement Therapy"[MeSH Terms] OR "Kidney Diseases"[MeSH Terms] OR "renal insufficienc*"[Title/Abstract] OR "kidney insufficienc*"[Title/Abstract] OR "Renal Transplantation"[Title/Abstract] OR "Kidney Grafting"[Title/Abstract] OR "Kidney Transplantation"[Title/Abstract]) AND (“exercise”[Mesh Terms] OR “physical activity”[Title/Abstract] OR “physical activities”[Title/Abstract] OR “physically active”[Title/Abstract] OR “exercise”[Title/Abstract] or "step per day"[Title/Abstract] OR "steps per day"[Title/Abstract] OR "step count"[Title/Abstract] OR "step/day"[Title/Abstract] OR "steps/day"[Title/Abstract] OR "step/d"[Title/Abstract] OR "steps/d"[Title/Abstract] OR "daily step"[Title/Abstract] OR "daily steps"[Title/Abstract])

Embase

('mortality'/exp OR "mortalities":ti,ab,kw OR "mortality":ti,ab,kw OR "fatality":ti,ab,kw OR "fatal":ti,ab,kw OR "death":ti,ab,kw) AND ('physical activity'/exp OR "physical activity":ti,ab,kw OR "physical activities":ti,ab,kw OR "physically active":ti,ab,kw OR "exercise":ti,ab,kw OR "step per day":ti,ab,kw OR "steps per day":ti,ab,kw OR "step count":ti,ab,kw OR "step/day":ti,ab,kw OR "steps/day":ti,ab,kw OR "step/d":ti,ab,kw OR "steps/d":ti,ab,kw OR "daily step":ti,ab,kw OR "daily steps":ti,ab,kw) AND ('kidney failure'/exp OR 'renal replacement therapy'/exp OR 'kidney disease'/exp OR "predialysis":ti,ab,kw OR "pre-dialysis":ti,ab,kw OR "kidney disease":ti,ab,kw OR "renal disease":ti,ab,kw OR "kidney failure":ti,ab,kw OR "renal failure":ti,ab,kw OR "renal insufficienc*":ti,ab,kw OR "kidney insufficienc*":ti,ab,kw OR "hemodialysis":ti,ab,kw OR "haemodialysis":ti,ab,kw OR "hemofiltration":ti,ab,kw OR "haemofiltration":ti,ab,kw OR "hemodiafiltration":ti,ab,kw OR "haemodiafiltration":ti,ab,kw OR "dialysis":ti,ab,kw OR "Renal Transplantation":ti,ab,kw OR "Kidney Grafting":ti,ab,kw OR "Kidney Transplantation":ti,ab,kw)

Web of Science

TS=(“physical activity” OR “physical activities” OR “physically active” OR “exercise” OR "step per day" OR "steps per day" OR "step count" OR "step/day" OR "steps/day" OR "step/d" OR "steps/d" OR "daily step" OR "daily steps") AND TS=("predialysis" OR "pre-dialysis" OR "kidney disease" OR "renal disease" OR "kidney failure" OR "renal failure" OR "hemodialysis" OR "haemodialysis" OR "hemofiltration" OR "haemofiltration" OR "hemodiafiltration" OR "haemodiafiltration" OR "dialysis" OR "renal insufficienc*" OR "kidney insufficienc*" OR "Renal Transplantation" OR "Kidney Grafting" OR "Kidney Transplantation") AND TS=(“mortalities” OR “mortality” OR “fatality” OR “fatal” OR “death”)

The Cochrane library

#	Search strategy
1	MeSH descriptor "Renal Insufficiency, Chronic" explode all trees
2	("Chronic Renal Insufficiency" OR "Chronic Kidney Insufficiency" OR "Chronic Kidney Disease" OR "Chronic Renal Disease" OR "Predialysis" OR "Pre-dialysis"):ti,ab,kw
3	("CKD" OR "CKF" OR "CRD" OR "CRF"):ti,ab,kw
4	("End-Stage Kidney" OR "End-Stage Renal" OR "Endstage Kidney" OR "Endstage Renal"):ti,ab,kw
5	("ESRD" OR "ESRF" OR "ESKD" OR "ESKF"):ti,ab,kw
6	MeSH descriptor "Renal Replacement Therapy" explode all trees
7	"Dialysis":ti,ab,kw
8	("Hemodialysis" OR "Haemodialysis" OR "Hemodiafiltration" OR "Haemodiafiltration" OR "HD):ti,ab,kw
9	"PD":ti,ab,kw
10	("Renal Transplantation" OR "Kidney Grafting" OR "Kidney Transplantation"):ti,ab,kw
11	"KTRs":ti,ab,kw
12	{or 1-11}
13	MeSH descriptor "exercise" explode all trees
14	("physical activity" OR “physical activities" OR "physically active" OR "exercise"):ti,ab,kw
15	("step per day" OR "steps per day" OR "step count" OR "step/day" OR "steps/day" OR "step/d" OR "steps/d" OR "daily step" OR "daily steps"):ti,ab,kw
16	{or 13-15}
17	MeSH descriptor "mortality" explode all trees
18	"mortalities" OR "mortality" OR "fatality" OR "fatal" OR "death"): ti,ab,kw
19	{or 17, 18}
20	{AND 12, 16, 19}
